# Effect of Aggressive Experience in Female Syrian Hamsters on Glutamate Receptor Expression in the Nucleus Accumbens

**DOI:** 10.3389/fnbeh.2020.583395

**Published:** 2020-11-23

**Authors:** Johnathan M. Borland, Ellen Kim, Samuel P. Swanson, Patrick E. Rothwell, Paul G. Mermelstein, Robert L. Meisel

**Affiliations:** Department of Neuroscience, University of Minnesota, Minneapolis, MN, United States

**Keywords:** social behavior, synaptic plasiticty, nucleus accumbens, metabotropic Glu receptors, female aggression

## Abstract

Our social relationships determine our health and well-being. In rodent models, there is now strong support for the rewarding properties of aggressive or assertive behaviors to be critical for the expression and development of adaptive social relationships, buffering from stress and protecting from the development of psychiatric disorders such as depression. However, due to the false belief that aggression is not a part of the normal repertoire of social behaviors displayed by females, almost nothing is known about the neural mechanisms mediating the rewarding properties of aggression in half the population. In the following study, using Syrian hamsters as a well-validated and translational model of female aggression, we investigated the effects of aggressive experience on the expression of markers of postsynaptic structure (PSD-95, Caskin I) and excitatory synaptic transmission (GluA1, GluA2, GluA4, NR2A, NR2B, mGluR1a, and mGluR5) in the nucleus accumbens (NAc), caudate putamen and prefrontal cortex. Aggressive experience resulted in an increase in PSD-95, GluA1 and the dimer form of mGluR5 specifically in the NAc 24 h following aggressive experience. There was also an increase in the dimer form of mGluR1a 1 week following aggressive experience. Aggressive experience also resulted in an increase in the strength of the association between these postsynaptic proteins and glutamate receptors, supporting a common mechanism of action. In addition, 1 week following aggressive experience there was a positive correlation between the monomer of mGluR5 and multiple AMPAR and NMDAR subunits. In conclusion, we provide evidence that aggressive experience in females results in an increase in the expression of postsynaptic density, AMPARs and group I metabotropic glutamate receptors, and an increase in the strength of the association between postsynaptic proteins and glutamate receptors. This suggests that aggressive experience may result in an increase in excitatory synaptic transmission in the NAc, potentially encoding the rewarding and behavioral effects of aggressive interactions.

## Introduction

Approximately one in five adults in the United States now suffer from a diagnosable mental disorder, such as depression, schizophrenia, anxiety disorder, and autism spectrum disorder (National Institute of Mental Health website: statistics). Importantly, common and overlapping traits key to many of these disorders are deficits in social processes (Henry et al., 2016; Novacek et al., 2016; Pegg et al., 2019; Snyder-Mackler et al., 2020). Hence, the rewarding properties of social interactions are critical for the expression of adaptive social behaviors (Darwin, 1859; Skinner, 1938; Trezza et al., 2011; Borland et al., 2017) and the development and maintenance of social relationships (Young and Wang, 2004; Krach et al., 2010; Borland et al., 2019b). These relationships can serve as a buffer to stress (Morrison et al., 2014; Smith and Wang, 2014) and reduce susceptibility to psychiatric and addictive disorders (Bodenmann and Randall, 2013; Nagy and Moore, 2017; Fett et al., 2019). Social interactions come in a variety of forms, including affiliation, aggression, parental and sexual behaviors, each with distinct molecular signatures (Young and Wang, 2004; Shahrokh et al., 2010; Albers, 2012; Staffend and Meisel, 2012; Staffend et al., 2014; Bredewold et al., 2015); yet due to the false belief that aggression is not a part of the normal repertoire of social behaviors displayed by females (Buss, 1961; Blanchard et al., 1988; Albers et al., 2002; Björkqvist, 2018), very little is known about the neural mechanisms mediating aggression in this sex (Been et al., 2019).

It is important to note that while previous studies of aggression have primarily focused on its maladaptive and destructive nature (Siever, 2008; Golden et al., 2019b), aggression under many circumstances can be advantageous (Crombach and Elbert, 2014; Wrangham, 2018). For example, assertiveness is key to protecting one’s rights and privileges (e.g., resist “bullying”). It is in this context that the positive rewarding effects of resisting intrusions can help develop a positive level of self-esteem that supports mental health (Morrison et al., 2014; Snyder-Mackler et al., 2016; Komori et al., 2019). Indeed, the pervasive expression of agonistic encounters, dominant-subordinate relationships, and ultimately social hierarchies across the animal kingdom is evidence of the beneficial, adaptive and rewarding properties of aggression (Meisel and Joppa, 1994; Abbott et al., 1998; Edwards and Spitzer, 2006; Pettinger et al., 2011; Albers, 2012; Gil et al., 2013). These adaptive processes are critical for resource allocation, reproductive success and thus survival for males and females (Digby and McLean Stevens, 2007; Nelson and Trainor, 2007; Lindenfors and Tullberg, 2011; Stockley and Bro-Jorgensen, 2011; Giebel et al., 2013).

The apex in the central nervous system for reward processing is the nucleus accumbens (NAc) (Koob, 1996; Salamone et al., 2009; Mohebi et al., 2019). Changes in the electrical transmission of neurons in this region underlie reward processing (Schultz et al., 1997; Kourrich et al., 2007). Using female Syrian hamsters, a well validated animal model for studying aggression in this sex (Drickamer and Vandenbergh, 1973), our lab recently showed that aggressive experience results in an increase of mature mushroom-like head dendritic spines on NAc medium spiny neurons (MSNs) (Staffend and Meisel, 2012). Although the molecular and physiological mechanisms mediating these postsynaptic changes are unknown, follow-up studies from our lab suggest PSD-95 and mGluR5 play a functional role in this synaptic structural plasticity (Peterson et al., 2015; Been et al., 2016; Gross et al., 2016). Furthermore, previous literature suggests that increases in mushroom head spines may be associated with increases in excitatory synaptic transmission and long-term potentiation; more specifically an increase in AMPAR expression (Malinow and Malenka, 2002; Muddashetty et al., 2007; Takahashi et al., 2009; Bencsik et al., 2019).

The glutamate system is universal in neuronal excitatory plasticity, consisting of a diverse assortment of ionotropic (AMPA/NMDA) and metabotropic (mGluR1-8) receptors with various kinetic and physiological properties (Reiner and Levitz, 2018; Goncalves et al., 2020). We postulate that increases in excitatory synaptic transmission in the NAc will likely encode the rewarding properties of aggression, similar to the effects of addictive drug exposure (Kourrich et al., 2007; Ebner et al., 2018; Turner et al., 2018a; Stagkourakis et al., 2020). To begin to develop this line of research, we investigated the effects of female aggressive experience on the expression of proteins associated with postsynaptic plasticity (PSD-95 and Caskin I) and excitatory synaptic transmission, AMPARs (GluA1, GluA2, and GluA4), NMDARs (NR2A and NR2B) and group I mGluRs (Gq-protein coupled monomer and dimer forms of mGluR1a and mGluR5) in the NAc. Furthermore, to gain insight into potential mechanisms mediating changes in postsynaptic density and excitatory synaptic transmission, we investigated the strength of associations between the various postsynaptic structure proteins and glutamate receptors analyzed, and potential effects of aggressive experience on these relationships. We hypothesized that aggressive experience results in the strengthening of the relationships between postsynaptic proteins and glutamate receptors in the NAc. In addition, based on previous literature (Been et al., 2016), we predict mGluR5 expression in particular to play a central role in this aggression mediated postsynaptic plasticity.

## Materials and Methods

### Subjects

Adult female (*n* = 36) and male Syrian hamsters (*n* = 24) (*Mesocricetus auratus*) were purchased from Charles River Laboratories (Wilmington, MA, United States) at approximately 60 days of age (120–130 g). Subject females were housed individually while intruder males were pair-housed in polycarbonate cages (females: 50.8 cm × 40.6 cm × 20.3 cm; males: 43.2 cm × 22.9 cm × 20.3 cm). These semi-natural housing arrangements maximize aggression in female subjects, while decreasing aggression in male intruders (Brain, 1972; Grelk et al., 1974; Gattermann et al., 2001; Ross et al., 2017). All animals were maintained on a reversed 14:10 light/dark photoperiod (lights off at 1300 h) and all behavioral testing occurred during the first 3 h of the dark phase. The animal room was maintained at a controlled temperature of 22°C and food and water were available *ad libitum*. Hamsters acclimated for 2 weeks before experiments. All animal procedures were carried out in accordance with the National Institutes of Health Guide for the Care and Use of Laboratory Animals (NIH Publications No. 80-23; revised 2011) and approved by the University of Minnesota Institutional Animal Care and Use Committee.

### Ovariectomy

To maximize aggressive behavior in females, circulating estradiol levels were maintained at low levels via bilateral ovariectomy (Meisel et al., 1988) under sterile conditions. Surgery was conducted under sodium pentobarbital anesthesia (Nembutal, 8.5 mg/100 g body weight, i.p.). Analgesic (Meloxicam, 1 mg/kg, s.c., Fort Dodge Animal Health, Overland Park, KS, United States) was administered for 3 days of post-operative pain management. Two subjects were preemptively sacrificed due to complications during surgery.

### Behavioral Testing and Scoring

Females were handled daily for at least 4 days prior to the first aggressive interaction session. A different adult male stimulus hamster was placed into a female subject’s home cage for five min each day for a five-day period. Each session was video recorded and later scored for two main criteria: (1) the latency to the subject’s first attack and (2) the total number of attacks. A two-step process was used to verify scoring (live scoring followed by video scoring). Attacks were operationally defined as the subject biting, or clearly attempting to bite, the intruder male. Intruder males were used at most every other day and for each test individual females were paired with a different male to minimize the likelihood that submission by the male would influence the behavior of the females (Huhman et al., 2003). No social interaction control females did not interact with males but did undergo the same handling and transport experiences as experimental groups.

### Tissue Collection

Twenty-four hours or 1 week following the fifth aggression test females were given an overdose of sodium pentobarbital (Beuthanasia-D, 0.25 ml i.p.,/animal, Schering, Union, NJ, United States) and sacrificed by rapid decapitation. Control subjects that did not experience social interaction also had tissue collected at these two time points. Coronal sections (approximately 1 – 2 mm) containing the NAc, caudate putamen (CPu) and medial prefrontal cortex (PFC) were taken with the aid of a brain matrix and bilateral tissue punches (1 mm diameter) were immediately collected from each area. NAc punches included the anterior commissure to bias the punches toward the NAc core, where we have previously found increases in dendritic spine density following aggressive experience (Staffend and Meisel, 2012). Dorsal medial CPu punches were taken to evaluate the regional specificity of the biochemical changes within the striatum following aggressive experience. Punches were flash-frozen and stored in an −80°C freezer.

### Tissue Preparation

To prepare tissue for Western blot analysis, flash frozen tissue was first transferred into 1.5 mL protein lysate tubes with metallic beads. 100 μl of ice-cold RIPA buffer (250 mM NaCl and 50 mM Tris pH 8) and 1 μl of HALT phosphatase and 1 μl of HALT protease inhibitors (Thermo Fisher, Waltham, MA, United States) were added to each lysate tube. Tissue samples were then homogenized in a bullet blender (Next Advance, Inc., Troy, NY, United States) for 6 min. 21 μl of detergent master mix (per 16 ml: 1 ml 100% triton, 10 ml of 10% sodium dodecyl sulfate (SDS) and 5 ml of 10% sodium deoxycholate) was then added to tubes with homogenized tissue. Samples were then rotated for 45 min with a sample mixer at 4°C. Samples were centrifuged at 4°C for 20 min (12,000 rpm). Finally, the supernatant was aspirated (∼80–100 μl) and placed in a fresh tube. Approximately 6 μl per sample was used for protein analysis and the rest was stored in a −80°C freezer until used for Western blots. Protein was quantified using Bio-Rad protein DC assay (Bio-Rad Laboratories, Berkeley, CA, United States). Samples were run in triplicate at 20, 10 and 6.7 dilution factors (1.0–3.0 μl of sample and 19.0–17.0 μl of 1% SDS). Standards were run in duplicate in eight serial dilutions of 1.5–0.05 mg/ml (Bio-Rad Laboratories).

### Western Blot

For each subject twenty or thirty μg of total protein were heated for 10 min on a 90°C hot plate. Samples were then loaded into a 12–15% polyacrylamide gradient gel in 1% SDS running buffer (Mini-PROTEAN TGX Precast Mini Gel, Bio-Rad Laboratories, 110 mV for ∼90 min). Proteins from the gel were then transferred to a nitrocellulose membrane (0.45 μm pore size, Bio-Rad Laboratories, Cat. # 1620115, 100 mV for 55 min). After transfer, membranes were then blocked in Odyssey Blocking Buffer (Li-Cor Biosiences, Lincoln, NE, United States) for 1 h. Membranes were then incubated overnight in primary antibodies against mouse anti-PSD-95 (1:1000; Invitrogen, Rockford, IL, United States, REF MA 1-045), rabbit anti-Caskin I (1:2000; Synaptic Systems, Göttingen, Germany, Cat. # 185 002), rabbit anti-GluA1 (1:2000; Abcam, Cambridge, MA, United States, ab31232), rabbit anti-GluA2 (1:1000; Cell Signaling, Danvers, MA, United States, Ref: 04/2019 5306S), rabbit anti-GluA4 (1:1000; Abcam, Cambridge, MA, United States, ab109431), rabbit anti-NR2A (1:500; Abcam, Cambridge, MA, United States, ab124913), rabbit anti-NR2B (1:1000; Abcam, Cambridge, MA, United States, ab65783), rabbit anti-mGluR1a (1:1000; Millipore, Billerica, MA, United States, 07-617), rabbit anti-mGluR5 (1:2000; Millipore, Billerica, MA, United States, AB5675) and mouse anti-GAPDH (1:10,000; Abcam, Cambridge, MA, United States, ab8245). The following day, the membranes were washed in TBST for six 3-min washes. Membranes were then incubated in a Li-Cor incubation box for 1 h in the appropriate secondary antibodies (goat anti-rabbit 680RD or 800CW IgG and goat anti-mouse 800CW IgG, 1:20,000, Li-Cor Biosciences, Lincoln, NE, United States) and then repeat washed in TBST six more times for 3 min each. Finally, membranes were imaged and analyzed using the Odyssey imaging system (Li-Cor Biosciences), as previous described (Been et al., 2016). Within each lane/subject, protein of interest was normalized to GAPDH. Within each gel, these relative expression values were then normalized to the average expression of subjects that did not experience social interaction (no social control) to reveal a fold change. Multiple proteins were assessed on the same gel: PSD-95 and mGluR5 (monomer and dimer), GluA1 and NR2A, GluA2 and NR2B, GluA4 and mGluR1a (monomer and dimer), and thus were normalized to the same GAPDH band (Supplementary Figure 2).

### Data Analysis

For behavioral analyses, mixed repeated-measures (RM) ANOVAs with Tukey’s *post hoc* tests were used to detect significant differences in aggressive behaviors between groups (tissue collected at 24 h versus 1 week after social experience) and across testing days (days 1 through 5), *p* < 0.05. For protein analyses, significant differences between groups were detected using one-way ANOVAs with LSD *post hoc* tests, *p* < 0.05 (Statistics are in Supplementary Tables 1–3). A Kruskal-Wallis test was performed to detect if aggressive experience resulted in a shift in the quality and strength of relationships between the different proteins in the NAc (categorized 1 through 7; strong ± 1.00 > *r* > 0.75, moderate 0.74 > *r* > 0.50, weak 0.49 > *r* > 0.25, none 0.24 > *r*), *p* < 0.05. Correlation matrices for each treatment examined the positive or negative relationships between each protein, *p* < 0.05 or *p* < 0.01. For correlation analyses fold change was normalized to 0. Data were analyzed using GraphPad Prism 8.3.0 for Windows. Two subjects were removed, as they did not display aggressive behaviors toward male intruders. Statistical outliers were removed using the ROUT method (maximum False Discovery Rate, *Q* = 1%). All data were examined to determine if the assumptions of parametric statistical tests were met, [normality (Bartlett’s test) and equal variance (Brown-Forsythe test)]. If assumptions were not met, Kruskal-Wallis (non-parametric) test was performed (along with Dunn’s *post hoc* tests). All tests were two-tailed, and results considered statistically significant if *p* < 0.05 unless specified otherwise. All data are presented as mean ± standard error of the mean.

## Results

### The Rewarding Properties of Five Consecutive Days of Aggressive Behavior

**FIGURE 1:**
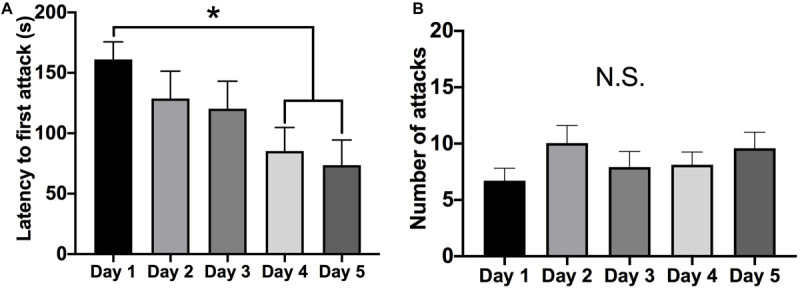
Aggressive behavior over five consecutive tests (5-min each). **(A)** There was a decrease in the latency to attack on the four and fifth test day compared to the first test day (*p* < 0.05). **(B)** There was no change in the average number of attacks over the five social interaction test days. (Days 1–5 *n* = 24). (*Indicates significant difference between groups, *p* < 0.05).

Female Syrian hamsters displayed a steady decrease in the latency to initiate an attack from the first to the fifth social behavior test session (mixed RM-ANOVA: *F* = 4.403, *p* = 0.005, *df* = 4, 92) (Figure 1A). Specifically, the latency to the first attack was shorter on the fourth (Tukey’s: *d* = 0.630, *p* = 0.038, *df* = 23) and fifth (Tukey’s: *d* = 0.664, *p* = 0.026, *df* = 23) days compared to the first testing day. However, there were no differences in the total number of attacks over the five test days (mixed RM-ANOVA: *F* = 1.554, *p* > 0.05, *df* = 4, 92) (Figure 1B). Following the five days of social interaction testing, brain tissue was collected for protein analysis either 24 h or 1 week later. There were no differences in either the latency to attack (mixed RM-ANOVA: *F* = 0.6688, *p* > 0.05, *df* = 1, 22) or the total number of attacks (mixed RM-ANOVA: *F* = 0.7522, *p* > 0.05, *df* = 1, 22) between subjects in which tissue was collected 24 h versus 1 week after behavior testing (Supplementary Figure 1).

### Effect of Aggressive Experience on Markers of Postsynaptic Morphology in the NAc

**FIGURE 2:**
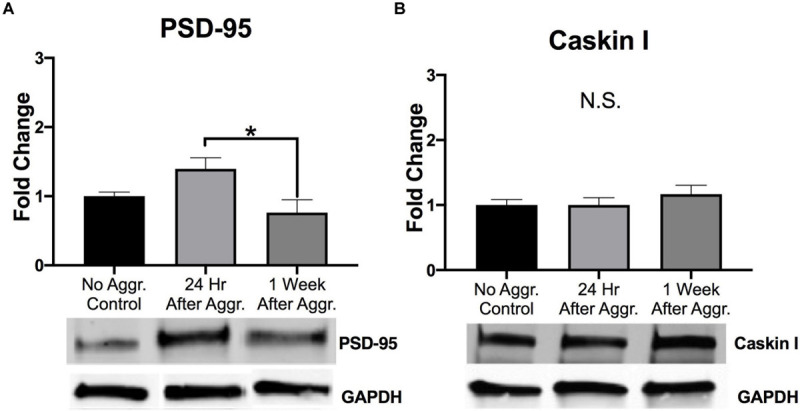
The effect of aggressive behavior on the expression of markers of postsynaptic plasticity in the NAc. **(A)** For subjects that had aggressive experience, there was an increase in PSD-95 expression in the NAc 24 h, compared to expression levels 1 week following aggression (*p* < 0.05). **(B)** Aggressive experience had no overall effect on Caskin I expression in the NAc. (Control *n* = 8–9, 24 h *n* = 11, 1 Week *n* = 9). (*Indicates significant difference between groups, *p* < 0.05).

Representative lanes for Western blot analysis can be found in Supplementary Figures 2A–E. Western blot analysis revealed that aggressive experience had a main effect on PSD-95 protein expression in the NAc (Kruskal-Wallis: *H* = 8.101, *p* = 0.017, *df* = 2) (Figure 2A). An increase in PSD-95 in the NAc 24 h after aggressive experience significantly decreased to that of control females 1 week later (Dunn’s test: *d* = 1.165, *p* = 0.013, *df* = 26). Finally, Caskin I protein expression was examined to assess if these changes in postsynaptic structural density may be linked to changes in specifically mushroom-like spines (Bencsik et al., 2019). There was no effect of aggressive experience on Caskin I expression in the NAc (one-way ANOVA: *F* = 0.668, *p* > 0.05, *df* = 2, 25) (Figure 2B).

### Effect of Aggressive Experience on AMPARs and NMDARs in the NAc

**FIGURE 3:**
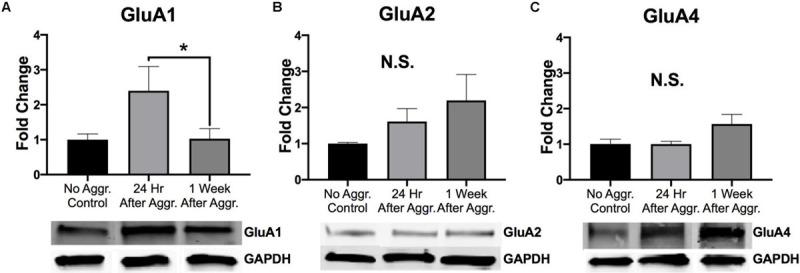
The effect of aggression on AMPAR subunit expression in the NAc. **(A)** Aggression resulted in an increase in GluA1 expression in the NAc 24 h following aggressive experience compared to 1 week following aggressive experience (*p* < 0.05). **(B)** There was no effect of aggressive experience on GluA2 expression. **(C)** There was no effect of aggressive experience on GluA4 expression. (Control *n* = 7–9, 24 h *n* = 10–11, 1 Week *n* = 9). (*Indicates significant difference between groups, *p* < 0.05).

**FIGURE 4:**
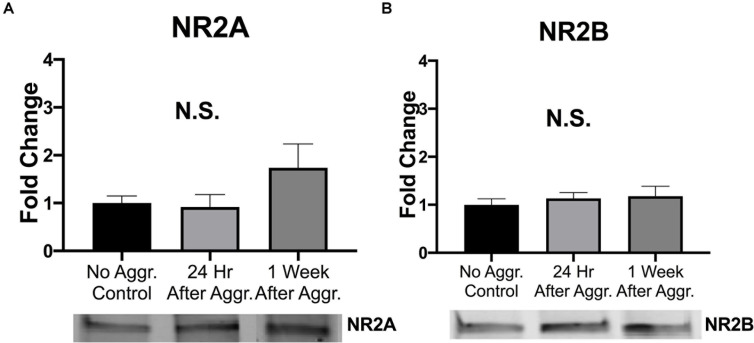
Aggression did not alter NMDAR subunit expression in the NAc. **(A)** Aggressive experience had no effect on overall NR2A expression in the NAc. **(B)** Aggressive experience had no effect on overall NR2B expression in the NAc. (Control *n* = 9, 24 h *n* = 11, 1 Week *n* = 9–10). (*p* > 0.05).

To examine if aggressive experience results in changes in AMPAR expression in the NAc, we examined expression levels of different AMPAR (GluA1, GluA2, and GluA4) subunits. There was a trend for GluA1 protein expression to be increased 24 h following aggressive experience (Dunn’s test: *d* = 1.042, *p* = 0.058, *df* = 25) compared to no social controls (Kruskal-Wallis: *H* = 5.572, *p* = 0.062, *df* = 2) (Figure 3A). This increase in GluA1 24 h following aggressive experience was attenuated 1 week later (Dunn’s test: *d* = 0.806, *p* = 0.033, *df* = 25). There was no main effect of aggressive experience on GluA2 expression in the NAc (one-way ANOVA: *F* = 1.308, *p* > 0.05, *df* = 2, 24) (Figure 3B). Similarly, there was no main effect of aggressive experience on GluA4 expression in the NAc (Kruskal-Wallis: *H* = 2.138, *p* > 0.05, *df* = 2), although the original parametric test (the data was not normally distributed) did detect a significant increase in GluA4 expression 1 week following aggressive experience (one-way ANOVA: *F* = 3.560, *p* = 0.043, *df* = 2, 26) compared to just 24 h following aggressive experience (Fisher’s LSD: *d* = 1.062, *p* = 0.032, *df* = 26) and compared to no social control subjects (Fisher’s LSD: *d* = 0.926, *p* = 0.025, *df* = 26) (Figure 3C). To examine if aggressive experience results in changes in NMDAR expression in the NAc, we examined expression levels of the NR2A and NR2B subunits. Aggressive experience had no effect on either NR2A (one-way ANOVA: *F* = 1.795, *p* > 0.05, *df* = 2, 27) or NR2B (one-way ANOVA: *F* = 0.709, *p* > 0.05, *df* = 2, 26) expression in the NAc (Figures 4A,B). In sum, there was an increase in GluA1 expression in the NAc 24 h versus 1 week following the final aggression experience.

### Effect of Aggressive Experience on mGluRs in the NAc

**FIGURE 5:**
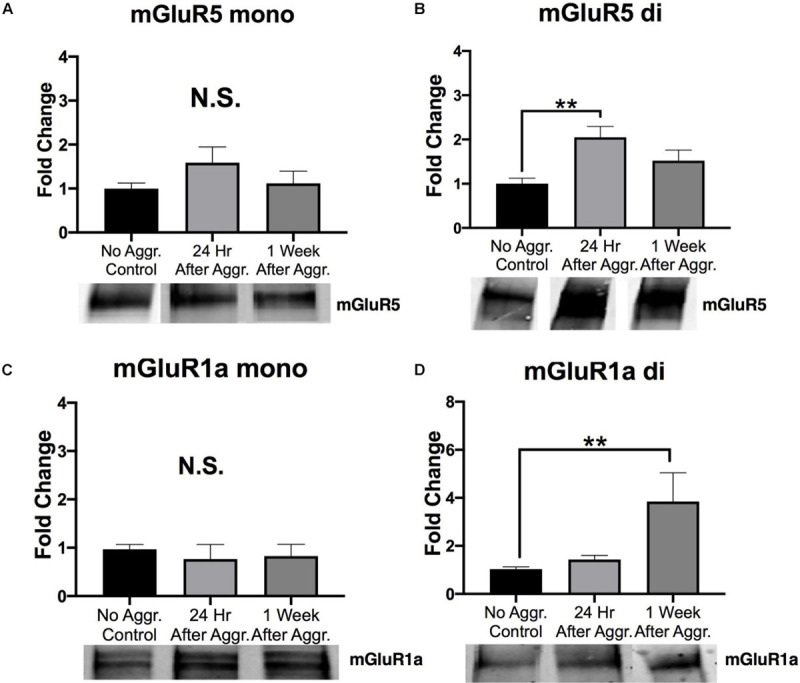
The effect of aggression on group I metabotropic glutamate receptor expression in the NAc. **(A)** There was no effect of aggressive experience on the expression of the monomer form (mono) of mGluR5 in the NAc. **(B)** There was an increase in the dimer form (di) of mGluR5 in the NAc 24 h following aggressive experience compared to subjects that did not have social experience (*p* < 0.05). **(C)** There was no effect of aggressive experience on the expression of the monomer form of mGluR1a. **(D)** There was an increase in the dimer form of mGluR1a in the NAc 1 week following aggressive experience compared to subjects that did not have social experience (*p* < 0.05). (Control *n* = 8-9, 24 h *n* = 9–12, 1 Week *n* = 9–10). (*Indicates significant difference between groups, *p* < 0.05; **p < 0.01).

To investigate if aggressive experience affects group I metabotropic glutamate receptor expression we examined expression levels of mGluR5 and mGluR1a, respectively. Aggressive experience resulted in an increase in the dimer (activated) form of mGluR5 in the NAc 24 h (Fisher’s LSD: *d* = 1.587, *p* = 0.002, *df* = 27) but not 1 week (*p* > 0.05) after aggressive experience as compared to control subjects that did not experience aggression (one-way ANOVA: *F* = 5.638, *p* = 0.009, *df* = 2, 27) (Figure 5B). Aggressive experience resulted in an increase in the dimer (activated) form of mGluR1a 1 week (Dunn’s test: *d* = 1.107, *p* = 0.007, *df* = 24), but not 24 h (*p* > 0.05) after aggressive experience compared to no social controls (Kruskal-Wallis: *H* = 7.305, *p* = 0.026, *df* = 2) (Figure 5D). For both of these receptors, these effects were observed only for the dimer form of the receptor. No changes were detected for the monomer forms of mGluR5 (one-way ANOVA: *F* = 1.204, *p* > 0.05, *df* = 2, 27) and mGluR1a (one-way ANOVA: *F* = 0.179, *p* > 0.05, *df* = 2, 26) (Figures 5A,C).

These changes in expression were selective to the NAc, as aggressive experience did not affect the overall expression levels of any of the proteins (PSD-95, GluA1, GluA2, GluA4, NR2A, NR2B, mGluR5, and mGluR1a) in tissue collected from the CPu or the PFC (one-way ANOVA: *F* = 0.037–2.099, *p* > 0.05, *df* = 2, 25–27) (Supplementary Figures 3, 4). Summary of NAc statistics can be found in Supplementary Table 1. Summary of CPu and PFC statistics can be found in Supplementary Tables 2, 3. In sum, aggressive experience results in an increase in the expression of PSD-95, GluA1 and the dimer forms of group I mGluRs in the NAc.

### Effect of Aggressive Experience on the Relationship and Strength of Association Between Postsynaptic Proteins and Glutamate Receptors in the NAc

**FIGURE 6:**
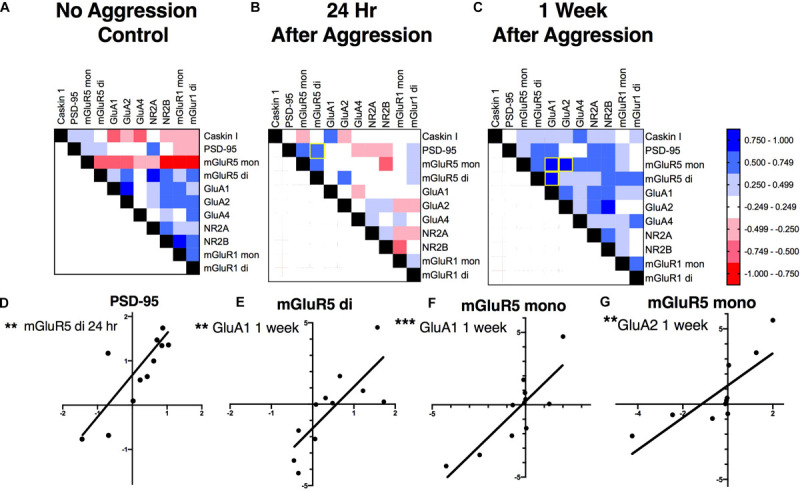
The effect of aggressive experience on the relationships between postsynaptic proteins and glutamate receptors in the NAc: correlation matrices. **(A–C)** One week following aggressive experience there is an increase in the strength of positive associations between the postsynaptic proteins and glutamate receptors analyzed (*p* < 0.001) (also see Figure 7). **(A)** Relationships between postsynaptic proteins and glutamate receptors in the NAc in subjects that did not have social experience. **(B)** Relationships in the NAc 24 h following aggressive experience. **(C)** Relationships in the NAc 1 week following aggressive experience. [Positive relationship: strong 1.00 > *r* > 0.75, moderate 0.74 > *r* > 0.50, weak 0.49 > *r* > 0.25 (blue); negative relationship: strong –1.00 > *r* > –0.75, moderate –0.74 > *r* > –0.50, weak –0.49 > *r* > –0.25 (red); no relationship 0.24 > *r* > –0.24 (white)]. **(D–G)** Representative correlations: **(D)** the expression of the dimer form of mGluR5 was positively correlated with PSD-95 expression 24 h following aggressive experience (*p* < 0.01), but not 1 week following aggressive experience, nor in subjects that did not have social experience (*p* > 0.05). **(E)** The mGluR5 dimer was positively correlated with GluA1 1 week following aggressive experience (*p* < 0.01). **(F)** The mGluR5 monomer was positively correlated with GluA1 and **(G)** GluA2 1 week following aggressive experience (*p* < 0.01). (**Indicates significant difference between groups, *p* < 0.01; ***p < 0.001).

**FIGURE 7:**
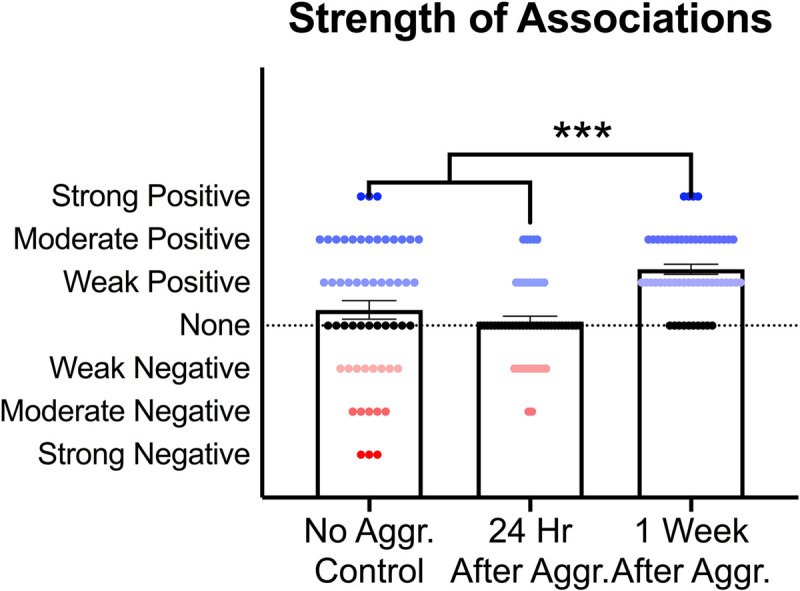
Effect of aggressive experience on the strength of relationships between postsynaptic proteins and glutamate receptors in the NAc. Aggressive experience resulted in an increase in the strength of positive associations between the proteins analyzed at 1 week compared to the strength of relationships 24 h following aggressive experience and compared to no social control subjects (*** = *p* < 0.001).

To investigate if these increases in PSD-95, GluA1 and mGluR5 dimer are correlated with each other, supporting a common mechanism of action, and if aggressive experience results in changes in the strength of the relationship between postsynaptic structural proteins and glutamate receptors, correlation matrices were calculated for each treatment condition in the NAc (Figures 6A–C). Aggressive experience resulted in a shift in the quality of the relationships between the different postsynaptic proteins and glutamate receptors analyzed (positive relationship: strong 1.00 > *r* > 0.75, moderate 0.74 > *r* > 0.50, weak 0.49 > *r* > 0.25; negative relationship: strong −1.00 > *r* > −0.75, moderate −0.74 > *r* > −0.50, weak −0.49 > *r* > −0.25; no relationship 0.24 > *r* > −0.24) (Kruskal-Wallis: *H* = 31.38, *p* < 0.001, *df* = 2). There was an increase in the strength of association between the different postsynaptic proteins and glutamate receptors analyzed 1 week following aggressive experience compared to no social controls (Dunn’s test: *d* = 0.747, *p* < 0.001, *df* = 162) and compared to 24 h following aggressive experience (Dunn’s test: *d* = 1.364, *p* < 0.001, *df* = 162) (Figure 7).

**FIGURE 8:**
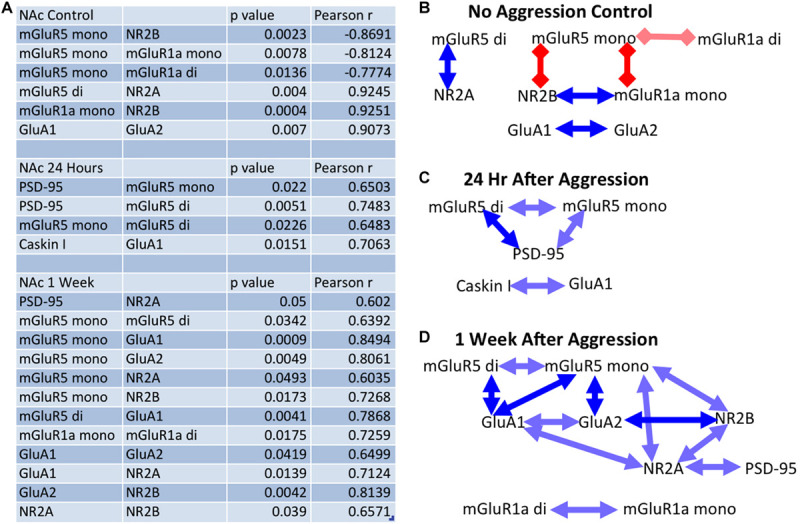
The effect of aggressive experience on the relationships between postsynaptic proteins and glutamate receptors in the NAc: significance table. **(A)** Table of significant correlations between proteins in the NAc in subjects that either did not encounter a social stimulus or subjects that had aggressive experience and had tissue collected either 24 h or 1 week later. **(B)** Significant relationships between proteins in the NAc in subjects that did not encounter a social stimulus. (Red square lines = negative/inverse relationship; blue arrow lines = positive/direct relationship; dark = *p* < 0.01, light = 0.01 < *p* < 0.05). At baseline: the mGluR5 dimer is positively correlated with NR2A, the mGluR5 monomer is negatively correlated with NR2B and the mGluR1a monomer and dimer, and mGluR1a monomer is positively correlated with NR2B expression. Finally, GluA1 is positively correlated with GluA2 expression. **(C)** Twenty-four hours following aggressive experience: the mGluR5 dimer is positively correlated with PSD-95 and the mGluR5 monomer, and PSD-95 is positively correlated with the mGluR5 monomer. Caskin I is positively correlated with GluA1. **(D)** One week following aggressive experience: the mGluR5 monomer is positively correlated with the mGluR5 dimer, GluA1, GluA2, NR2B, and NR2A, the mGluR5 dimer is positively correlated with GluA1, GluA1 is positively correlated with GluA2 and NR2A, GluA2 is positively correlated with NR2B, NR2A is positively correlated with NR2B and PSD-95, and the mGluR1a dimer is positively correlated with its monomer form.

More specifically, at baseline (no aggressive experience), the mGluR5 dimer was positively correlated with NR2A (Pearson correlation coefficient: *r* = 0.925, *p* = 0.004, *df* = 7), the mGluR5 monomer was negatively correlated with NR2B (*r* = −0.869, *p* = 0.002, *df* = 7) and the mGluR1a monomer (*r* = −0.812, *p* = 0.008, *df* = 7) and dimer (*r* = −0.777, *p* = 0.014, *df* = 7), and the mGluR1a monomer was positively correlated with NR2B (*r* = 0.925, *p* < 0.001, *df* = 7) expression. Finally, the GluA1 subunit was positively correlated with GluA2 expression [*r* = 0.907, *p* = 0.007, *df* = 7] (Figure 8B). Twenty-four hours following aggressive experience: the mGluR5 dimer was positively correlated with PSD-95 (*r* = 0.748, *p* = 0.005, *df* = 10, Figure 6D) and the mGluR5 monomer (*r* = 0.648, *p* = 0.023, *df* = 10), and PSD-95 was positively correlated with the mGluR5 monomer (*r* = 0.650, *p* = 0.022, *df* = 10). Finally, Caskin I was positively correlated with GluA1 (*r* = 0.706, *p* = 0.015, *df* = 9) (Figure 8C). One week following aggressive experience: the mGluR5 monomer was positively correlated with the mGluR5 dimer (*r* = 0.639, *p* = 0.034, *df* = 9), GluA1 (*r* = 0.849, *p* = 0.001, *df* = 9, Figure 6F), GluA2 (*r* = 0.806, *p* = 0.005, *df* = 8, Figure 6G), NR2A (*r* = 0.604, *p* = 0.049, *df* = 9) and NR2B (*r* = 0.727, *p* = 0.017, *df* = 8), the mGluR5 dimer was positively correlated with GluA1 (*r* = 0.787, *p* = 0.004, *df* = 9, Figure 6E), GluA1 was positively correlated with GluA2 (*r* = 0.650, *p* = 0.042, *df* = 8) and NR2A (*r* = 0.712, *p* = 0.014, *df* = 9), and the GluA2 subunit was positively correlated with NR2B (*r* = 0.814, *p* = 0.004, *df* = 8). Finally, the NR2A subunit was positively correlated with NR2B (*r* = 0.657, *p* = 0.039, *df* = 8) and PSD-95 (*r* = 0.602, *p* = 0.050, *df* = 9), and the mGluR1a dimer was positively correlated with its monomer form (*r* = 0.726, *p* = 0.018, *df* = 8) (Figure 8D). In conclusion, aggressive experience results in a positive synchronization in the amount of expression between almost all the different glutamate receptors analyzed, and in particular, the mGluR5 monomer was central.

## Discussion

The overall goal of this study was to begin to characterize responses of components of glutamate neurotransmission following aggressive experience in a female model. We previously investigated the functional necessity of mGluR5 activity on parameters associated with repeated aggressive experience in females (Been et al., 2016). Those results made us take a step back to provide an extensive analysis of protein changes in glutamate receptors and postsynaptic proteins following aggressive experience.

### The Rewarding Properties of Aggressive Experience in Female Syrian Hamsters

There is an accumulating body of literature investigating the neural mechanisms underlying the rewarding properties of aggressive behavior (Fish et al., 2005; Couppis and Kennedy, 2008; Gray et al., 2015; Golden et al., 2017, 2019a; Aleyasin et al., 2018; Panasiuk et al., 2019). However, these studies have been both primarily focused on the role of dopaminergic neurotransmission and heavily biased toward only studies of males. Thus, the overarching goal of this study was to bridge these gaps by (1), investigating the role of glutamatergic transmission and synaptic plasticity in aggression reward processing, and (2), focusing on the neural mechanisms mediating aggression in females. In sum, in this study, female aggressive experience resulted in dynamic changes in the expression of postsynaptic proteins including glutamate receptors in the NAc.

We first wanted to provide evidence that aggressive experience can affect parameters of future encounters in females. Female Syrian hamsters displayed a steady decrease in the latency to initiate an offensive attack against an intruding male over the five consecutive days of testing. In contrast, there was no change in the overall number of attacks. This supports the hypothesis that aggressive behavior comprises a feed-forward process involving reward processing, increasing behavioral efficiency (Thorndike, 1905; Skinner, 1938; Meisel and Joppa, 1994). It is theorized that increases in the strength of synaptic transmission underlie the rewarding consequences of stimuli (Kasai et al., 2003; Malenka, 2003; Ebner et al., 2018; Turner et al., 2018a). Thus, this study was the first to investigate if aggression might result in increases in molecular markers of postsynaptic plasticity and excitatory synaptic transmission and increases in the strength of the associations between these postsynaptic proteins and glutamate receptors in the NAc (Pitchers et al., 2012; Bredewold et al., 2015; Garcia-Pardo et al., 2019; Zoicas and Kornhuber, 2019).

### Overall Changes in Molecular Markers of Excitatory Transmission in the NAc

In line with previous literature, aggressive experience resulted in a transient increase in PSD-95 expression in the NAc 24 h following aggressive experience (Staffend and Meisel, 2012; Been et al., 2016). This effect was attenuated 1 week later. Thus, aggressive behavior is likely resulting in an increase in postsynaptic structural density in the NAc. To assess if these postsynaptic changes are specifically related to changes in mature mushroom-like spines, we investigated Caskin I protein expression. Interestingly, we did not detect any changes in Caskin I protein expression. Thus, the postsynaptic structural changes may have been independent of changes specifically in mushroom-like spines or our experimental procedure may not have been selective or sensitive enough to detect more subtle changes in expression (Bencsik et al., 2019). We next investigated if these changes in markers of postsynaptic structural density correlate with changes in excitatory synaptic transmission in the NAc.

Similar to PSD-95, there was an increase in the expression of the GluA1 subunit in the NAc 24 h following aggressive experience. The AMPA subunit GluA1 is highly associated with and implicated in the formation of mature mushroom-like spines and excitatory synaptic plasticity (Diering and Huganir, 2018). Interestingly, although GluA2 subunit membrane trafficking is implicated in calcium impermeability and spine stabilization (Henley and Wilkinson, 2013; Briand et al., 2016; Diering and Huganir, 2018), no significant differences in GluA2 were detected. Suggesting that the increases in AMPAR are calcium permeable, as has been seen with cocaine experience (Wolf and Tseng, 2012). There was a trend for an increase in GluA2 expression 1 week following aggression experience. Furthermore, although GluA4 has been primarily only implicated in neurodevelopment, we also observed a non-significant trend for increased expression in GluA4 1 week following aggressive experience, suggesting that it may play a role in the long-lasting behavioral effects of aggression (Huupponen et al., 2016; Luchkina et al., 2017; Atanasova et al., 2019).

Gating of AMPAR membrane trafficking and plasticity is synonymous with activation (Ca2 + influx) of NMDARs. Thus, we next investigated the effects of aggressive experience on the expression of the NR2A and NR2B NMDAR subunits. No overall changes in NR2A and NR2B expression were detected in the NAc. However, the NMDA receptor can still play a critical role in AMPAR trafficking, synaptic plasticity and long-term potentiation without actual changes in expression levels (i.e., second messenger effects) (Fox et al., 2006; Chang et al., 2015). AMPARs cluster in domains that surround a main NMDAR domain at the postsynaptic density (Goncalves et al., 2020). Further studies should investigate if aggressive experience results in changes in the activity, i.e., phosphorylation of NMDARs in the NAc, or if systemic treatment of an NMDAR antagonist blocks the molecular, morphological and behavioral effects of aggression in females. Similar to the GluA2 and GluA4 subunits, there was a trend for an increase in the expression of the NR2A subunit 1 week following aggression experience.

Activation, trafficking and translation of metabotropic glutamate receptors (a Gq-protein coupled receptor) have also been shown to play a critical role in the regulation of synaptic plasticity, AMPAR and NMDAR expression; and thus long-term potentiation and learning and memory (Menard and Quirion, 2012). There was a transient increase in the expression of the dimer form of mGluR5 24 h, but not 1 week, following aggressive experience. Interestingly, there was an increase in the dimer form of mGluR1a 1 week, but not 24 h, following aggressive experience. There were no significant changes in the overall expression levels of the monomer forms of mGluR5 and mGluR1a in the NAc. Collectively, aggressive experience likely results in an increase in postsynaptic structural density (potentially mushroom-like spines: evidence provided by an increase in GluA1), and excitatory synaptic transmission in the NAc (Staffend and Meisel, 2012; Meyer et al., 2014; Been et al., 2016; Diering and Huganir, 2018).

An important note, even though the mGluR5 and mGluR1a subunits may form heterodimers with each other (Pandya et al., 2016). There was an increase in mGluR5 dimer, but not mGluR1a dimer, 24 h following aggression, with an increase in the mGluR1a dimer 1 week following aggression, without any increase in mGluR5 dimer expression. These dissociations support the interpretation of homodimerization of the mGluRs. A recent study suggests that allosteric interactions and not heterodimerization may underlie the interactions between mGluR5 and mGluR1a (Kammermeier and McCullock, 2020).

All of the significant effects observed in the NAc were exclusive to the NAc, as there were no overall changes in any of the proteins analyzed in the CPu or PFC. However, it is not known if the increases in PSD-95, GluA1 and mGluR5 observed in the NAc are correlated with each other, supporting a common or linked mechanism of action; or not, supporting independent mechanisms of action. Thus, this study then investigated associations between these postsynaptic structural proteins and various glutamate receptors. Furthermore, this study was the first to investigate if aggressive experience results in changes in the strength of associations between postsynaptic proteins and glutamate receptors.

### Synaptic Protein and Glutamate Receptor Relationships in the NAc, Effects of Aggressive Experience

Aggressive experience resulted in an increase in the strength of relationships between the different postsynaptic proteins and glutamate receptors analyzed in the NAc. At baseline, without aggressive experience, there was an inverse relationship between mGluR5 and mGluR1a. Furthermore, mGluR5 was positively associated with the NR2A subunit, while mGluR1a was positively associated with the NR2B subunit of the NMDA receptor. Collectively, these results further provide evidence for an inverse role between mGluR1a and mGluR5 (Gross et al., 2016; Turner et al., 2018b), a reciprocal role between NR2A and NR2B in long-term potentiation and long-term depression (Massey et al., 2004; Bartlett et al., 2007), and differences in NMDAR function and learning and memory (Bar-Shira et al., 2015). In other words, these results provide support for the theory that mGluR1a and mGluR5 activation have opposite roles in NAc synaptic morphology, neuronal transmission and behavior (Dewing et al., 2007; Staffend and Meisel, 2012; Staffend et al., 2014; Been et al., 2016; Gross et al., 2016; Pitchers et al., 2016) through differences in their association with specific NMDAR subunits (Yashiro and Seki, 2017; Zoicas and Kornhuber, 2019). In agreement with previous literature, there was also a positive correlation in the expression of GluA1 and GluA2 (Diering and Huganir, 2018).

Twenty-four hours following aggressive experience, when the expression of PSD-95 and mGluR5 dimer and GluA1 are all increased in the NAc, the expression of both the mGluR5 dimer and monomer are positively correlated with PSD-95 expression, and the GluA1 subunit is positively correlated with Caskin I expression. These correlations are not observed in females that did not have aggressive experience. Collectively, PSD-95, Caskin I, GluA1, and mGluR5 expression are all associated with increases in mature mushroom-like spines and increases in excitatory postsynaptic transmission (Malinow and Malenka, 2002; Muddashetty et al., 2007; Takahashi et al., 2009; Peterson et al., 2015; Been et al., 2016; Bencsik et al., 2019). Thus, the increases in PSD-95, GluA1, and mGluR5 observed in the NAc are correlated together, further supporting a common or linked mechanism of action (Goncalves et al., 2020).

Finally, 1 week following aggressive experience, there was a distinct increase in the strength of positive correlations between almost all of the proteins analyzed, a “positive synchronization.” In particular, the monomer form of mGluR5 was positively correlated with the expression of the dimer form of mGluR5, the GluA1, GluA2, NR2A, and NR2B subunits; and more generally, all these glutamate receptor subunits, along with PSD-95, become positively associated with each other. Previous studies have also implicated mGluR5 in the NAc in synaptic plasticity and social reward processing (Stoker et al., 2012; Been et al., 2016; Gross et al., 2018). However, this is the first study to directly correlate the monomer form of mGluR5 with markers of postsynaptic structure (PSD-95) and multiple ionotropic glutamate receptors (GluA1, GluA2, NR2A, and NR2B) following social experience. This is particularly interesting considering that the dimer form of mGluRs is widely regarded as the active, functional or membrane trafficked form of the receptor, while the monomer form is regarded as the inactive or cytoplasm trafficked form of the receptor (Robbins et al., 1999; Lum et al., 2016). However, the monomer mGluR5 may also be a more direct measure of the translation of mGluR5. Thus, the broad correlation of the mGluR5 monomer with the various ionotropic glutamate receptor subunits 1 week following aggressive experience may be an indication of a common epigenetic messenger linked to mGluR5 activation regulating the translation of AMPARs, NMDARs and mGluR5 (Menard and Quirion, 2012; Sweatt, 2016). While the overall increases in the mGluR5 dimer may be an indication of increased postsynaptic membrane trafficking of mGluR5. Collectively, these findings implicate the group I mGluR5 in the regulation of excitatory postsynaptic plasticity in the NAc for female aggressive experience.

This is the first study to our knowledge that has comprehensively assessed and demonstrated that aggressive experience in females results in dynamic changes in the expression of both ionotropic and metabotropic glutamate receptors, and that these changes are also tightly associated and coupled with changes in postsynaptic proteins. These changes in markers of postsynaptic plasticity and glutamate receptors likely represent increases in excitatory input that could mediate a heightened response in synaptic transmission, potentially encoding the rewarding effects of aggressive experience (increased efficiency in the display of aggressive behavior) (Weiss et al., 1992; Kasai et al., 2003; Kourrich et al., 2007; Stagkourakis et al., 2020). In this regard, aggressive experience in male mice results in a persistent increase in excitatory synaptic transmission of estrogen receptor expressing neurons in the ventrolateral ventromedial hypothalamus (Stagkourakis et al., 2020). Future studies should investigate if this structural plasticity and increases in glutamate receptor expression in the NAc translates to an increase in excitatory sensitivity and transmission of MSNs that may mediate a heightened response to future activation in females (Beloate and Coolen, 2017; Turner et al., 2018a; Venniro et al., 2018).

### Translational Implications

For this study we were specifically focused on elucidating the molecular mechanisms mediating aggressive behavior in females, as previous studies of aggression are heavily biased toward males. There is evidence to suggest that there may be both conserved and divergent molecular mechanisms underlying aggression reward between males and females (Terranova et al., 2016; Borland et al., 2019a). There is also strong evidence that there are both conserved (Wallace et al., 2008; Hedges et al., 2009; Lobo et al., 2013; Pitchers et al., 2013; Aleyasin et al., 2018; Hamilton et al., 2018; Heshmati et al., 2018; Golden et al., 2019a) and divergent (Staffend and Meisel, 2012; Staffend et al., 2014) mechanisms underlying different social behaviors and animal species. We hypothesize that the recruitment of the glutamate system in the NAc for encoding social reward is conserved between males and females and across different species (Lei et al., 2020; Stagkourakis et al., 2020). Indeed, it has been previously postulated that glutamate terminals in the NAc regulate the coordinated activity of the oxytocin (via presynaptic OTRs on dorsal raphe 5-HT terminals) and serotonin system (via presynaptic 5-HT1b receptors on glutamate terminals) in gating social reward in male mice (Dolen et al., 2013; Walsh et al., 2018; Nardou et al., 2019). Furthermore, Stagkourakis et al. (2020) demonstrated that aggressive experience in male mice results in long-lasting increases in the excitatory synaptic transmission of ventrolateral ventromedial hypothalamus estrogen receptor expressing neurons. However, no studies to date have investigated if changes in glutamate receptor expression/plasticity in the NAc are critical for social reward processing.

Thus, our study is the first to provide direct evidence that plasticity in glutamate receptor protein expression may indeed be directly involved in social reward processing and synaptic plasticity in female Syrian hamsters. More specifically, we identify PSD-95, GluA1, and mGluR5 in the immediate, but perhaps not long-term effects of social reward in the NAc. The mGluR1a and perhaps GluA2, GluA4, and NR2A receptor subunit may underlie the long-term encoding of social reward. Furthermore, we provide evidence that there may be a coordinated recruitment of these different glutamate receptor components for encoding increases in excitatory synaptic transmission in MSNs in the NAc. Thus, future studies should investigate if the coordinated recruitment of these glutamate components in the NAc observed for female Syrian hamsters are also linked with the long-term potentiation mechanisms observed for social reward in male mice (Dolen et al., 2013; Stagkourakis et al., 2020). Once there is a sufficient representation of neurobiological studies of female aggression that issues of sex differences and commonalities in specific mechanisms should be explored.

Regarding comparisons with other natural reward, we have published a series of studies with sexual experience in female Syrian hamsters suggesting a casual link for synaptic plasticity and morphology in the NAc in the rewarding properties of sex behavior (Hedges et al., 2009; Been et al., 2013; Staffend et al., 2014; Moore et al., 2019). Coolen’s group has published similar findings for male sex behavior in rats (Pitchers et al., 2010, 2013). Thus, a collection of evidence, strongly suggest that the changes in the markers of synaptic structure and glutamate receptor expression in the NAc play a critical role in the rewarding effects of naturally occurring social behaviors in rodents.

## Conclusion

In conclusion, this study supports the hypothesis that aggression results in an increase in excitatory postsynaptic plasticity and transmission in the NAc and that this excitatory transmission may underlie the rewarding properties of social interactions in females (Been et al., 2016). Furthermore, there is potential that these social processes interact with the same neural mechanisms and pathways involving addictive behavior and drugs of abuse (Kourrich et al., 2007; Venniro et al., 2018). Thus, social interaction, or more specifically assertiveness, may serve as an effective and drug free treatment strategy for drug addiction and psychiatric disorders like depression (Francis and Lobo, 2017; Nagy and Moore, 2017; Komori et al., 2019). Syrian hamsters are the ideal model for studies of the reinforcing properties of aggressive interactions because like primates, both males and females, naturally and reliably establish dominance relationships (Digby and Kahlenberg, 2002; Stockley and Bro-Jorgensen, 2011; Been et al., 2019). Furthermore, recent studies of social reward in Syrian hamsters have had significant translational application to studies in humans (Terranova et al., 2017; Borland et al., 2019b). As previous studies of the mechanisms regulating social reward have been heavily biased toward males, the overall goal of this study was to further our understanding of the molecular mechanisms regulating the rewarding properties of aggression in females. This is one of the first studies to implicate excitatory postsynaptic plasticity and potentially long-term potentiation in the NAc in the rewarding properties of aggressive behavior in females.

## Disclosure

The content is solely the responsibility of the authors and does not necessarily represent the official views of the National Science Foundation and National Institutes of Health. The authors report no biomedical financial interests or potential conflicts of interest.

## Data Availability Statement

The raw data supporting the conclusions of this article will be made available by the authors, without undue reservation.

## Ethics Statement

The animal study was reviewed and approved by the University of Minnesota Institutional Care and Use Committee.

## Author Contributions

JB, SS, and EK conducted all experiments. JB analyzed the data and wrote the first draft of this manuscript. RM supervised all aspects of this study including the data analysis and contributed significantly in the experimental design, and preparation and editing of the manuscript. SS, EK, PR, and PM also provided vital input in the experimental design, and the preparation and editing of the manuscript.

## Conflict of Interest

The authors declare that the research was conducted in the absence of any commercial or financial relationships that could be construed as a potential conflict of interest.
