# Supplementary material for: Effect of Aggressive Experience in Female Syrian Hamsters on Glutamate Receptor Expression in the Nucleus Accumbens
Source: Front Behav Neurosci. 2020 Nov 23;14:583395. doi: 10.3389/fnbeh.2020.583395 (PMC7719767; doi:10.3389/fnbeh.2020.583395)

**Supplementary Material**


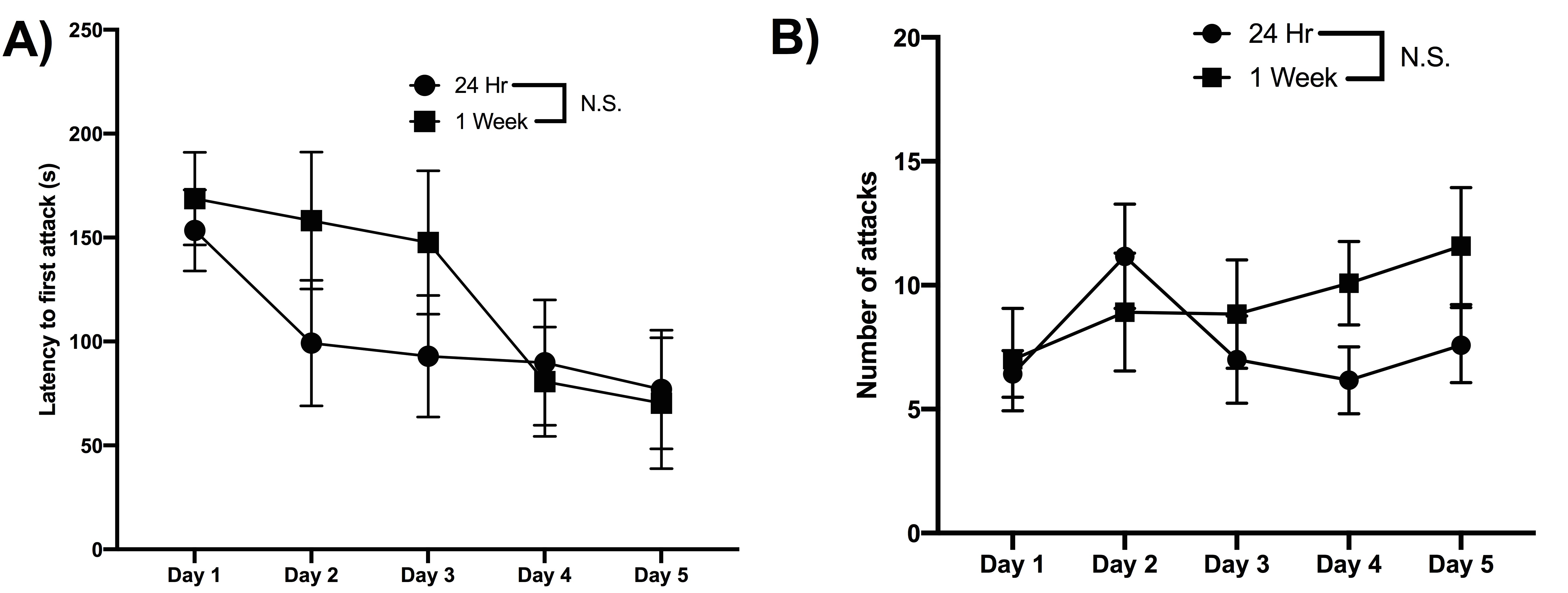


Supplementary Figure 1: Aggressive behavior between subjects in which tissue was collected 24 hours versus 1 week following aggressive experience. **(A)** There was no difference in the latency to the first attack between subjects in which tissue was collected 24 hours versus 1 week following aggressive experience (*p*>0.05). **(B)** There was no difference in the average number of attacks between subjects in which tissue was collected 24 hours versus 1 week following aggressive experience (*p*>0.05).

Supplementary Figure 2: Representative protein blots. Note: multiple proteins were assessed on the same gel. (**A)** NR2B and GluA2 protein blot. **(B)** NR2A and GluA1 protein blot. (**C)** PSD-95 and mGluR5 (monomer and dimer) protein blot. **(D)** mGluR1a (monomer and dimer) and GluA4 protein blot. (**E)** Caskin I protein blot. Increasing the amount of protein loaded into a lane (20μg vs 30μg) resulted in an increase in the expression intensity of GAPDH (*t*=6.237, *p*<0.001, *df*=62).


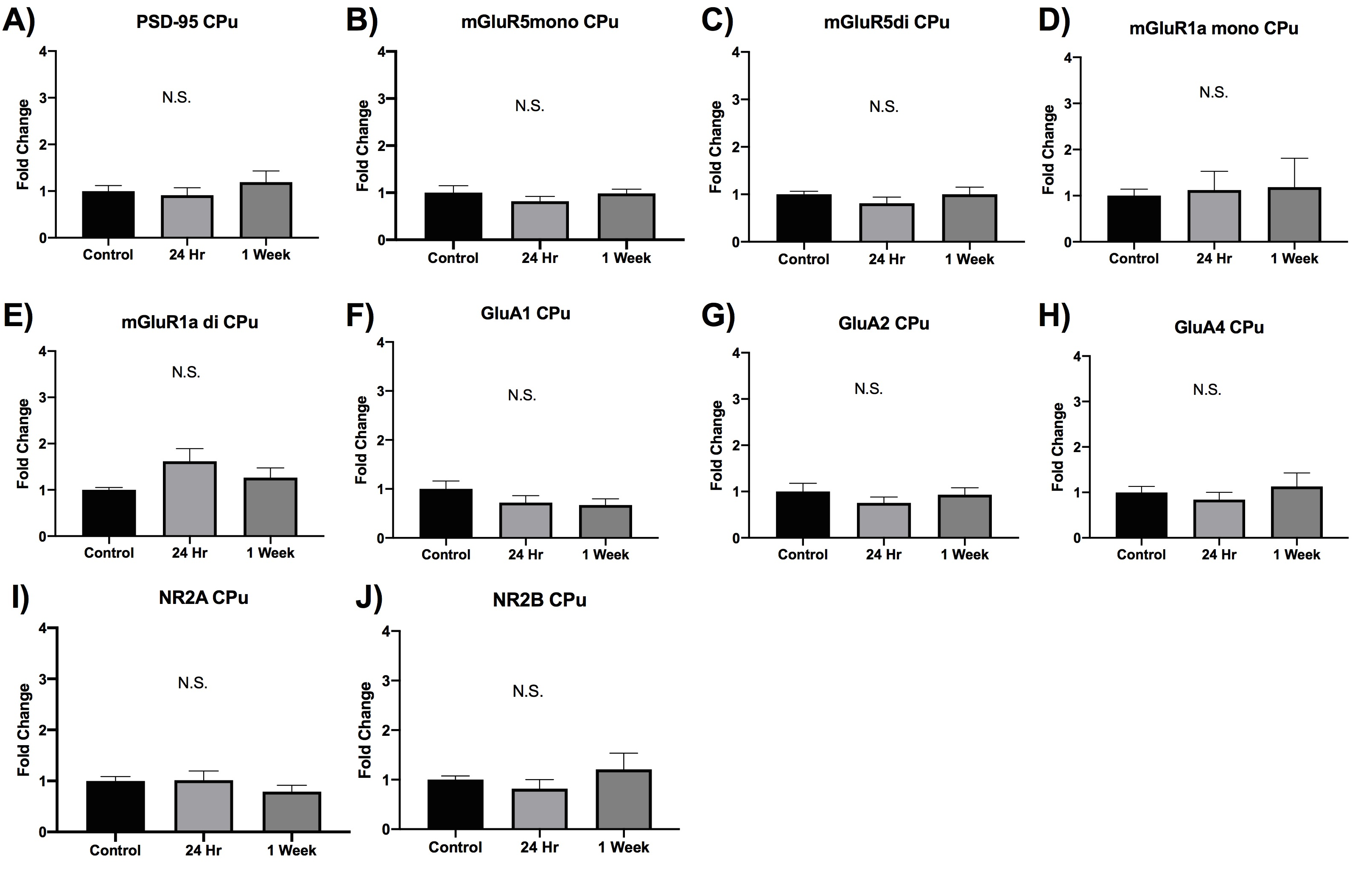


Supplementary Figure 3: Effect of aggressive experience on protein expression in the CPu. **(A-J)** Aggressive experience had no overall effect on the expression levels of PSD-95, mGluR5 monomer, mGluR5 dimer, mGluR1a monomer, mGluR1a dimer, GluA1, GluA2, GluA4, NR2A nor NR2B expression in the CPu (*p*>0.05). Statistics can be found in Supplementary Table 2.


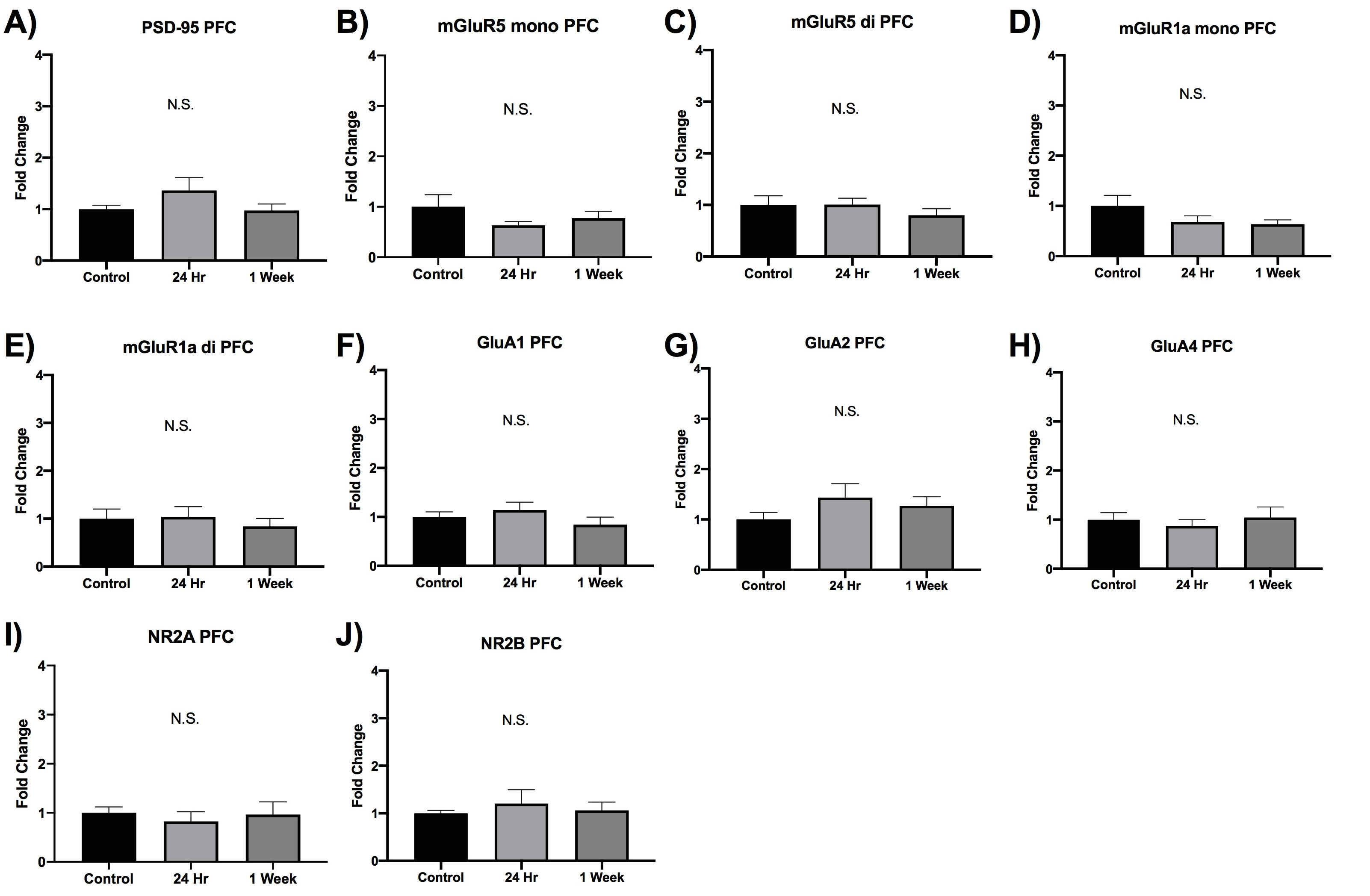


Supplementary Figure 4: Effect of aggressive experience on protein expression in the PFC. **(A-J)** Aggressive experience had no overall effect on the expression levels of PSD-95, mGluR5 monomer, mGluR5 dimer, mGluR1a monomer, mGluR1a dimer, GluA1, GluA2, GluA4, NR2A nor NR2B expression in the PFC (*p*>0.05). Statistics can be found in Supplementary Table 3.

Supplementary Table 1: One-way ANOVAs of relative protein expression in the NAc in subjects that did not experience social interaction (control) and subjects that had aggressive experience and had tissue collected either 24 hours or 1 week following aggressive experience. (Bold = significance, di = dimer, mono = monomer, KW = Kruskal-Wallis test). (* = *p*<0.05; ** = *p*<0.01; *** = *p*<0.001).

Supplementary Table 2: One-way ANOVAs of relative protein expression in the CPu in subjects that did not experience social interaction (control) and subjects that had aggressive experience and had tissue collected either 24 hours or 1 week following aggressive experience. (Di = dimer, mono = monomer). (* = *p*<0.05; ** = *p*<0.01; *** = *p*<0.001).

Supplementary Table 3: One-way ANOVAs of relative protein expression in the PFC in subjects that did not have social interaction experience (control) and subjects that had aggressive experience and had tissue collected either 24 hours or 1 week following aggressive experience. (Di = dimer, mono = monomer). (* = *p*<0.05; ** = *p*<0.01; *** = *p*<0.001).

**Original Gels: (A)** Caskin I, GAPDH; **(B)** NR2B, GluA2, GAPDH; **(C)** mGluR5, PSD-95, GAPDH; **(D)** NR2A, GluA1, GAPDH; **(E)** mGluR1a, GluA4, GAPDH.

A) Caskin I


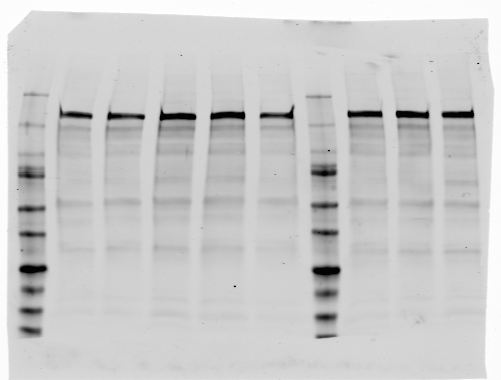


A) GAPDH


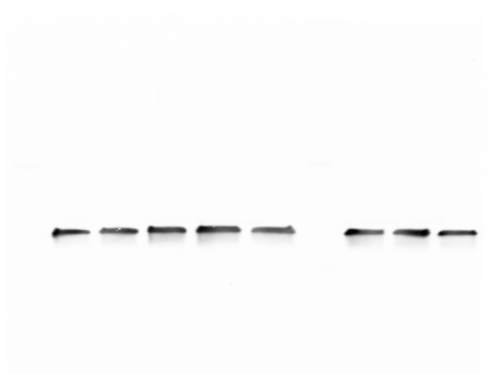


B) NR2B, GluA2 and GAPDH


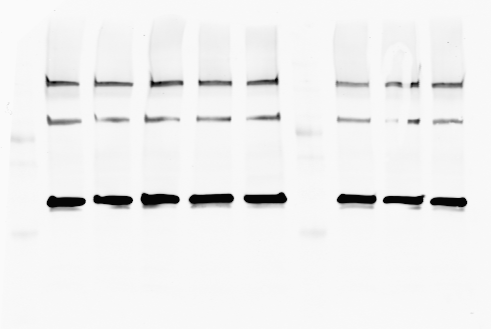


C) mGluR5 dimer, monomer, PSD-95 and GAPDH


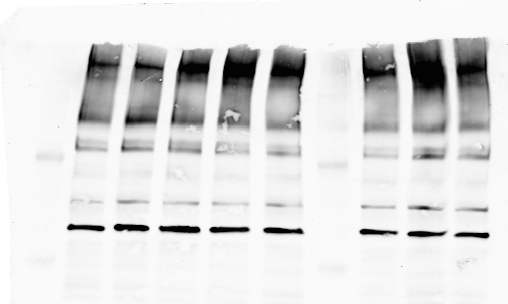


D) NR2A and GluA1


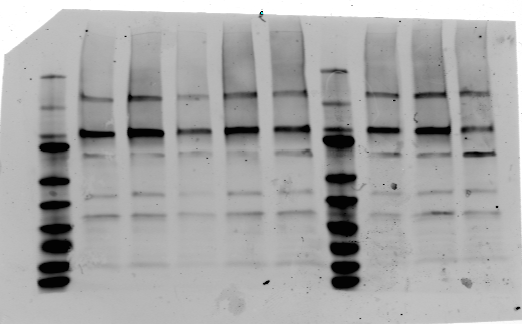


D) GAPDH


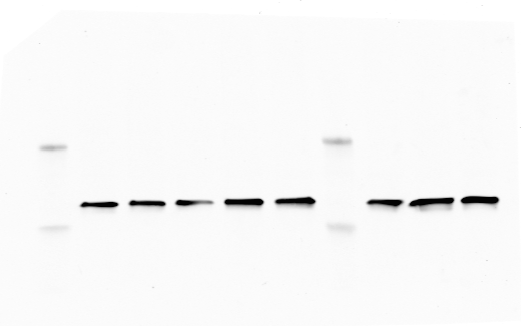


E) mGluR1a dimer, monomer and GluA4


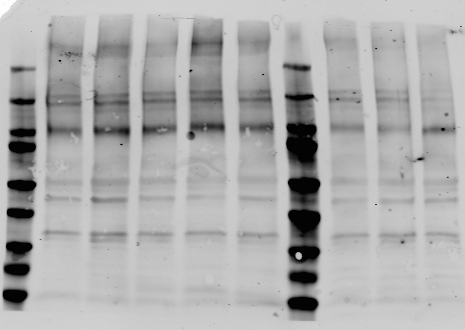


E) GAPDH


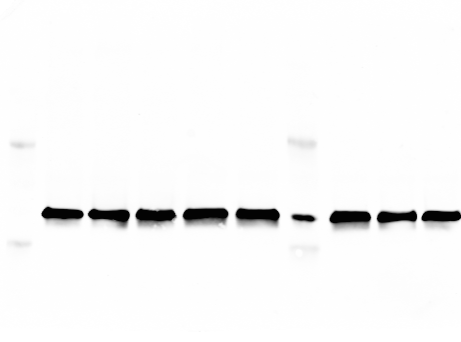

Supplement: Supplementary file 1 [file Table_1.DOCX]
